# Using bivalve chronologies for quantifying environmental drivers in a semi-enclosed temperate sea

**DOI:** 10.1038/s41598-018-23773-w

**Published:** 2018-04-03

**Authors:** M. Peharda, I. Vilibić, B. A. Black, K. Markulin, N. Dunić, T. Džoić, H. Mihanović, M. Gačić, S. Puljas, R. Waldman

**Affiliations:** 1 Institute of Oceanography and Fisheries, Split, Croatia; 2 Marine Science Institute, University of Texas at Austin, Port Aransas, TX USA; 3 Instituto Nazionale di Oceanografia e di Geofisica Sperimentale, Trieste, Italy; 4 Faculty of Science, University of Split, Split, Croatia; 5 Centre National de Recherches Météorologiques, Météo-France, Toulouse, France

## Abstract

Annual growth increments formed in bivalve shells are increasingly used as proxies of environmental variability and change in marine ecosystems, especially at higher latitudes. Here, we document that well-replicated and exactly dated chronologies can also be developed to capture oceanographic processes in temperate and semi-enclosed seas, such as the Mediterranean. A chronology is constructed for *Glycymeris pilosa* from a shallow embayment of the northern Adriatic and extends from 1979 to 2016. The chronology significantly (p < 0.05) and positively correlates to winter sea surface temperatures, but negatively correlates to summer temperatures, which suggests that extreme winter lows and extreme summer highs may be limiting to growth. However, the strongest and most consistent relationships are negative correlations with an index of the Adriatic-Ionian Bimodal Oscillating System (BiOS) for which positive values indicate the inflow of the ultraoligotrophic Eastern Mediterranean waters to the Adriatic. In contrast, the substantial freshwater flows that discharge into the Adriatic do not correlate to the bivalve chronology, emphasizing the importance of remote oceanographic processes to growth at this highly coastal site. Overall, this study underscores the potential of bivalve chronologies to capture biologically relevant, local- to regional-scale patterns of ocean circulation in mid-latitude, temperate systems.

## Introduction

A significant limitation in establishing the role of climate variability on ecological functioning in marine ecosystems is the lack of long-term observational records, particularly for biological phenomena, the lengths of which rarely exceed thirty years^1^. To address this issue, techniques borrowed from tree-ring science have been increasingly applied to growth-increment widths in the hard parts of marine bivalves and fish. Resulting chronologies are well replicated, annually resolved, and exactly dated to fully capture population-wide growth anomalies across a range of timescales^2–4^. These records may be readily compared with observational records to establish long-term climate-growth relationships and interrelationships among a diverse array of biological phenomena^5,6^. Moreover, growth-increment chronologies, especially from bivalves, can greatly exceed the length of instrumental records, often by an order of magnitude. For example, chronology of *Arctica islandica* has been constructed over 1357 years^7^. As such, they provide an opportunity to reconstruct historical ranges of climate variability, quantify pre-industrial baselines, and when combined with terrestrial proxies, describe interactions between the atmosphere and ocean^7–9^.

Ecological and environmental changes in marine ecosystems occur at local, regional and global scales and are a consequence of the interplay of multiple stressors. Semi-enclosed seas, such as the Mediterranean, are especially vulnerable due to a high surface to volume ratio^10^, and intense pressures from diverse human activities^11,12^. The Mediterranean is a temperate sea with much higher salinities than the adjacent Atlantic Ocean because evaporation exceeds new water inputs^13^. For that reason, circulation is characterized by the inflow of less saline Atlantic water, which becomes increasingly saline and subsides at the Gulf of Lion, the Adriatic, the Aegean and the Levantine convection sites to intermediate and deep layers^14,15^.

The northern Ionian Sea is a major driver of Eastern Mediterranean thermohaline circulation, including the Adriatic Sea. Cyclonic or anticyclonic rotation of the gyre in the northern Ionian Sea advects water masses to the Adriatic Sea that are: (i) of lower salinity and temperature and richer in nutrients from the Western Mediterranean, or (ii) of higher salinity and temperature and poor in nutrients from the Eastern Mediterranean^16^. These water masses influence dense water formation processes in the Adriatic Sea^17^, which in turn influence the circulation regimes in the northern Ionian Sea^18^. This oscillatory pattern called Adriatic-Ionian Bimodal Oscillating System^18^ (BiOS) drives decadal-scale thermohaline oscillations in the Adriatic Sea^19,20^ and the Eastern Mediterranean^21^.

These decadal oscillations affect Adriatic biogeochemical properties^22,23^ and even fisheries^24^. However, coastal regions, particularly the shallow northern Adriatic, are also characterized by strong freshwater influx (Po River, average discharge of about 1500 m^3^/s)^25^. Therefore, they have been considered separated from the overall Adriatic circulation and relatively more strongly affected by local than regional processes^26,27^. Yet, recent studies on physical properties^20^, long-term ocean carbon measurements^28^ and bivalve chronologies in the middle Adriatic^29^ indicate that Adriatic-wide processes and remote forcing may dominate over local forcing, even in coastal areas.

**Figure 1:**
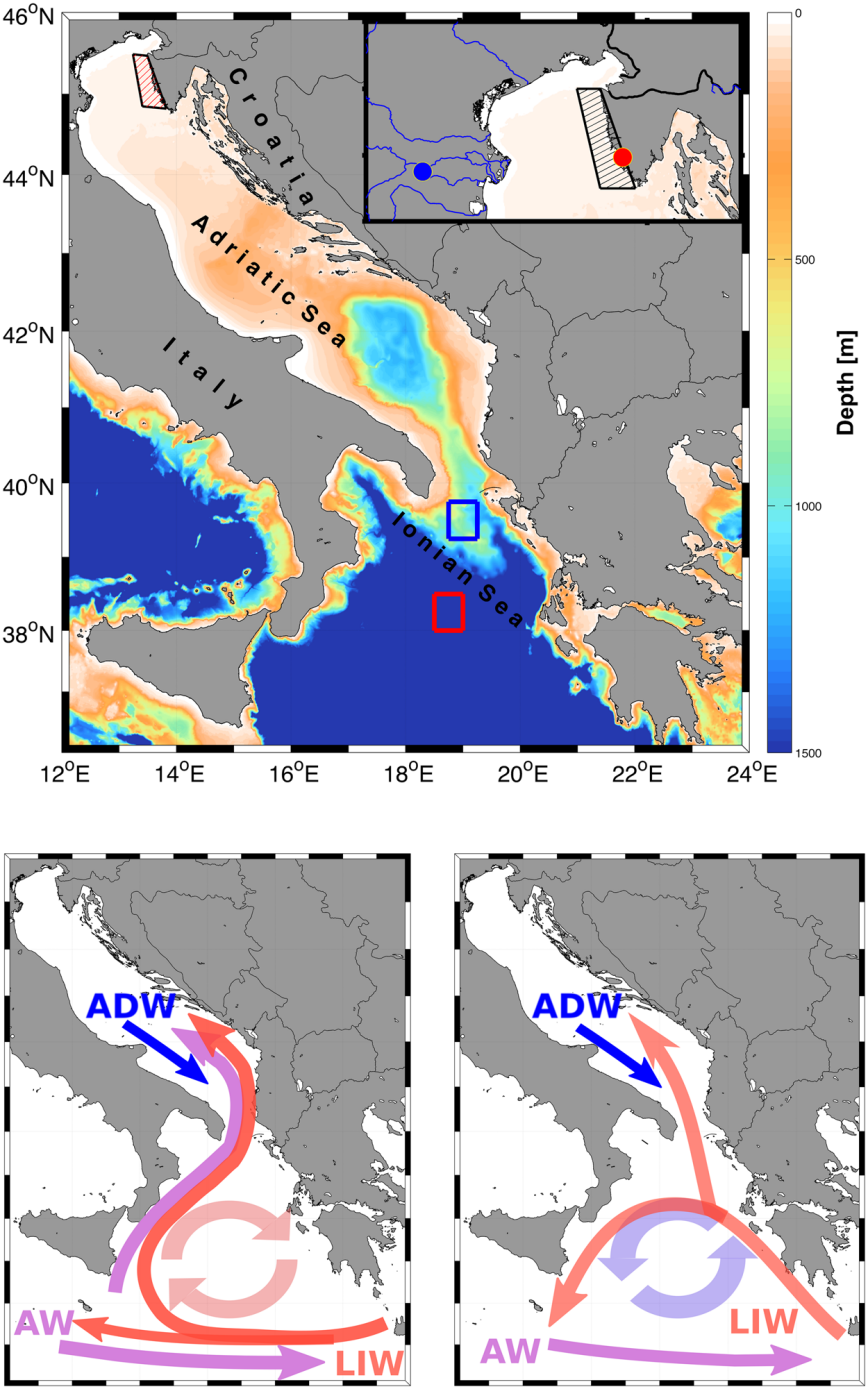
Map of the Adriatic-Ionian area indicating the sampling *Glycymeris pilosa* site along the Western Istria shoreline (red dot), Pontelagoscuro limnological station measuring the Po River discharge PO (blue dot), and areas used for constructing environmental variables: area over which ERA interim products sea surface temperature (SST), downward net heat flux (NHF), total precipitation (TP), air temperature at 2 m (T2M) are averaged (red mesh polygon); area over which NEMOMED12 ocean climate model variables, sea surface temperature (SSTN) and salinity (SSSN), are averaged (black mesh polygon). Blue and red rectangles denote the averaging areas in the northern Ionian Sea from which data on differences in absolute dynamic topography (ADT) are derived. ADW – Adriatic Deep Water, AW – Atlantic Water, LIW – Levantine Intermediate Water. The figure has been created using MATLAB 2014a (www.mathworks.com), Inkscape 0.92 (www.inkscape.org) and GIMP 2.8.16 (www.gimp.org) software.

Here, we develop a well-replicated, exactly dated chronology from the annual growth-increment widths of *G*. *pilosa* along the eastern coast of the northern Adriatic Sea (Fig. 1). This chronology provides a long time series to test the hypothesis that local forcing (such as rivers, precipitation, heat fluxes) is relatively more important than remote forcing (through advection of remote water masses) to biological processes of shell growth. In doing so, the chronology is related to a number of local and remote physical drivers extracted from a variety of long-term datasets: atmosphere reanalysis, ocean climate models and satellite altimetry. Determining the relative role of these processes will provide baseline information of use for marine resources management and understanding the role of climate variability and change on Adriatic ecosystems.

## Results

### Chronology construction

**Figure 2:**
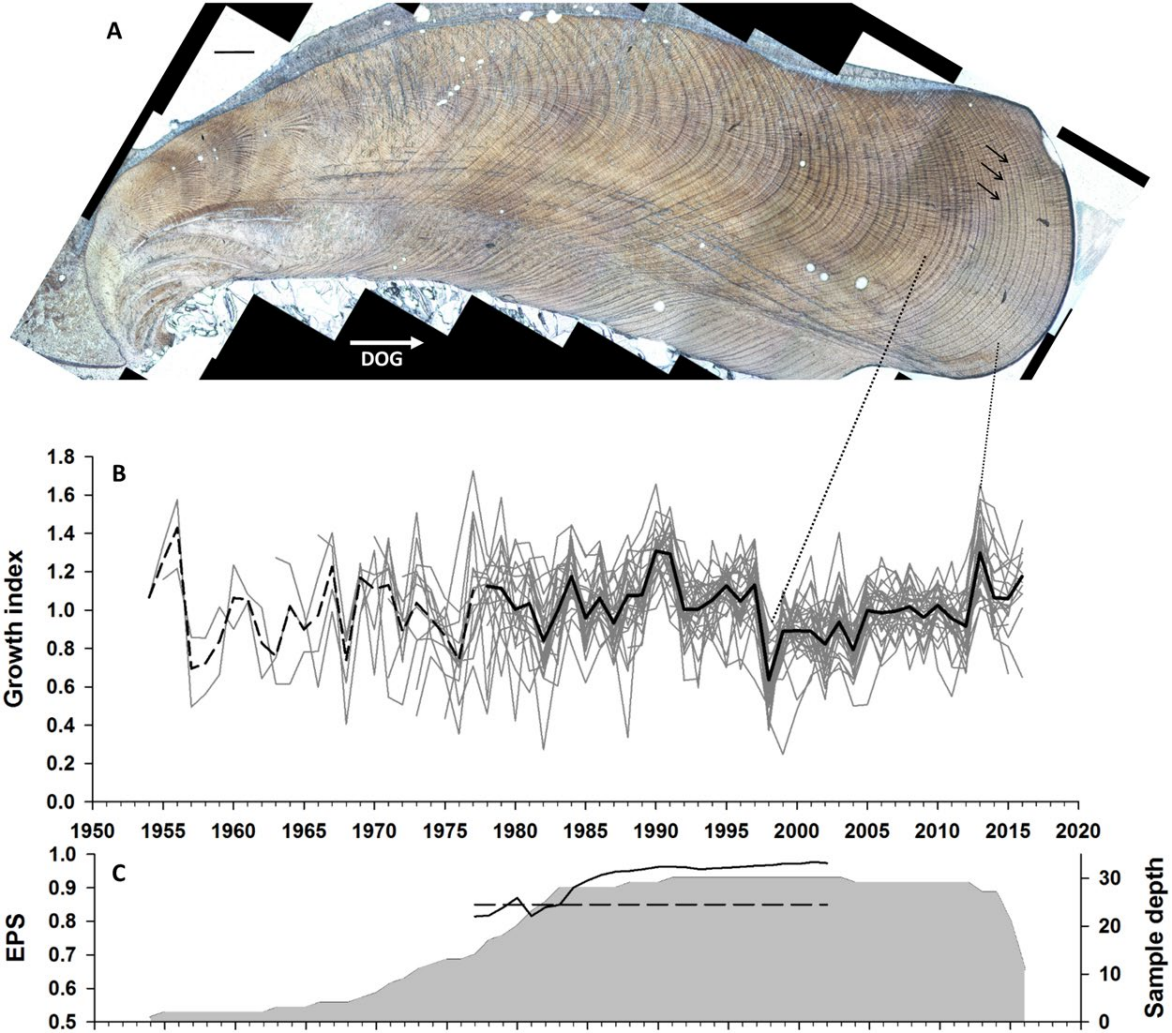
*Glycymeris pilosa* chronology. (**A**). Acetate peel of specimen S3P99 (scale bar 500 µm), direction of growth (DOG) indicated by white arrow, growth lines indicated by black arrows, (**B**). Individual detrended growth series (1954–2016) and their average (1979 statistically robust chronology in black, prior to 1979 in dashed line), (**C**). Sample depth (denoting number of samples, grey shading area) and Expressed Population Signal (EPS) values computed in a 30-year window with indicated arbitrary value of EPS value ≥ 0.85.

Individuals ranged in age from 35 to 72 years (N = 30, mean ± st.dev. = 55.8 ± 9.3 years), 11 of which were older than 60 years. The first growth increments (earliest ontogenetically) did not have clear boundaries, so measurements started from the 3^rd^ increment or later (mean ± st.dev. = 14.3 ± 8.85 year). Shells had a strongly synchronous growth pattern among individual specimens with conspicuously wide increments in 2013, 1991, and 1990 and narrow increments in 2004, 1998, 1982 and 1976. Series intercorrelation, representing the mean correlation between each detrended time series and the average of others, was 0.615, while average mean sensitivity was 0.183. The oldest measured growth increment dates back to year 1954, resulting in 63 years of data through 2016 (Fig. 2). Mean segment length was 39.9 years, and over 10 individual time series were measured over the period spanning 1973 to 2016. Running Expressed Population Signal calculated over a 30-year window with 29-year overlap indicated that the chronology was robust after 1979. Synchronous shell growth was noted also prior to this period with conspicuously narrow growth increments corresponding to years 1976 and 1968.

### Correlations to environmental drivers

**Figure 3:**
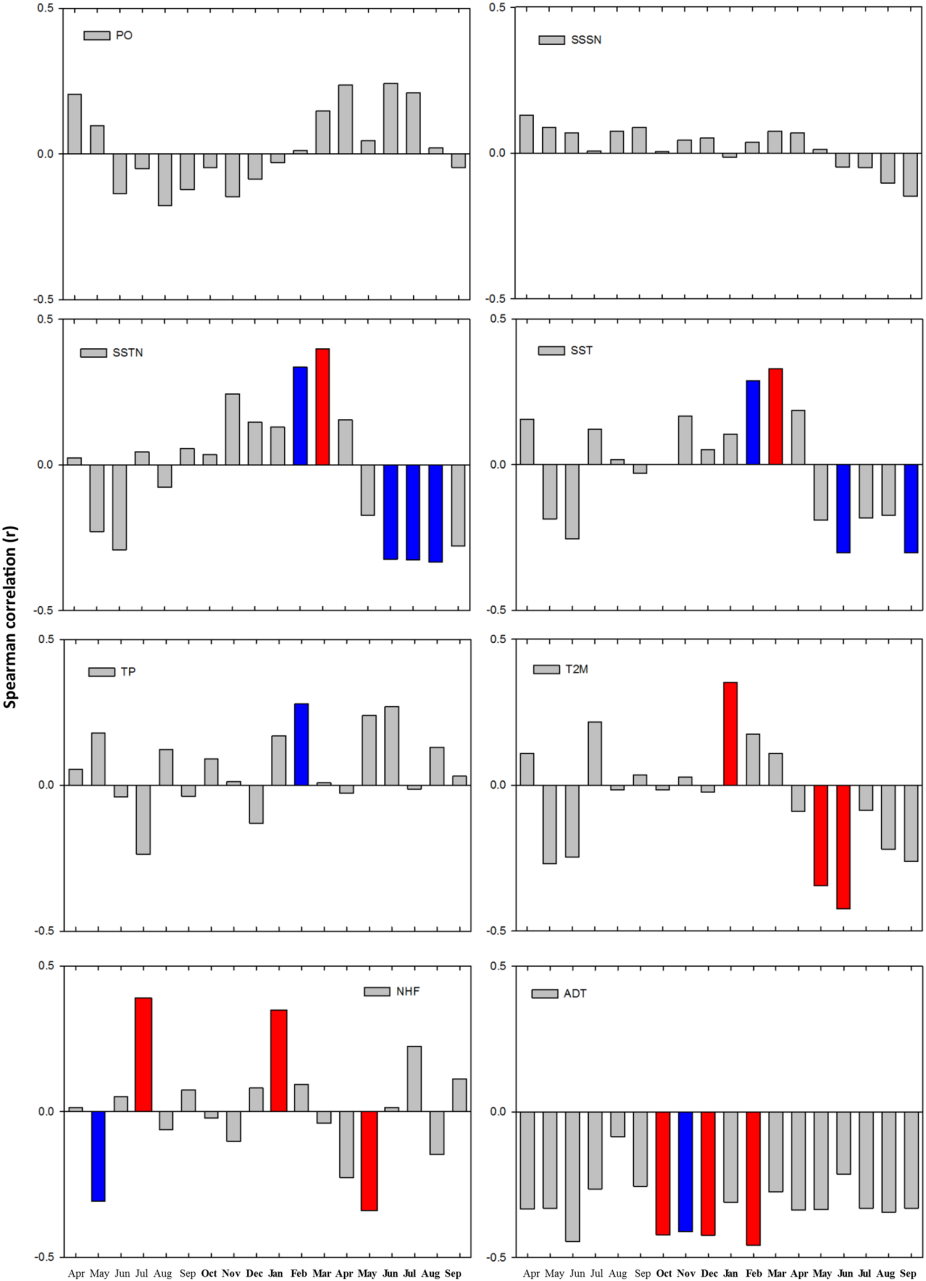
Spearman correlations between chronology and environmental variables. Red bars denote significance at 95%, blue bars significance at 90%. Months October to September refer to the year in which chronology has been computed (indicated in bold), while months April to September indicate months preceding the growth increment deposition. Po river discharge (PO), salinity from NEMOMED12 (SSSN), sea surface temperature from NEMOMED12 (SSTN), sea surface temperature from ERA (SST), total precipitation from ERA (TP), air temperature at 2 m from ERA (T2M), downward net heat flux from ERA (NHF), and monthly absolute dynamic topography (ADT).

Po River discharge (PO) and surface salinity (SSSN) are not correlated with the shell growth chronology in any month of the year (Fig. 3). The chronology correlates with precipitation (TP) for just one month of the year (February), and only at the 90% level (r = 0.28, p = 0.093, Fig. 3). Poor relationships with precipitation or salinity are typical across most of the Adriatic, as evidenced by a correlation analysis with gridded data (not shown), although there are some small patches of significant correlations at 95% between chronology and SSSN or TP. Sea surface temperature did, however, relate more strongly to the chronology, and with results that are generally consistent across the temperature variables considered in this study. Indeed, sea surface temperatures from the ECMWF reanalysis and NEMOMED12 (SSTN) model positively correlate to the chronology in the winter months (February (r = 0.34, p = 0.056) and March (r = 0.40, p = 0.022)), but negatively correlate in the summer months (May through September, r values between −0.33 and −0.32, p values between 0.058 and 0.067) (Fig. 3). A similar correlation pattern is evident for air temperature at 2 m, though the seasonality is somewhat lagged with peak relationships early in the winter and early in the summer (Fig. 3). In contrast, correlations with downward heat flux (NHF) are not as consistent with the other variables, especially in months from the prior year (Fig. 3). However, the balance of the evidence suggests that relatively higher winter temperatures and relatively lower summer temperatures appear to be associated with favourable growth conditions. These tendencies are corroborated in spatial correlations between the chronology and thermal environmental fields (T2M, SST, SSTN, NHF) in which growth is positively related to winter temperatures and negatively related to summer temperatures (data not shown).

**Figure 4:**
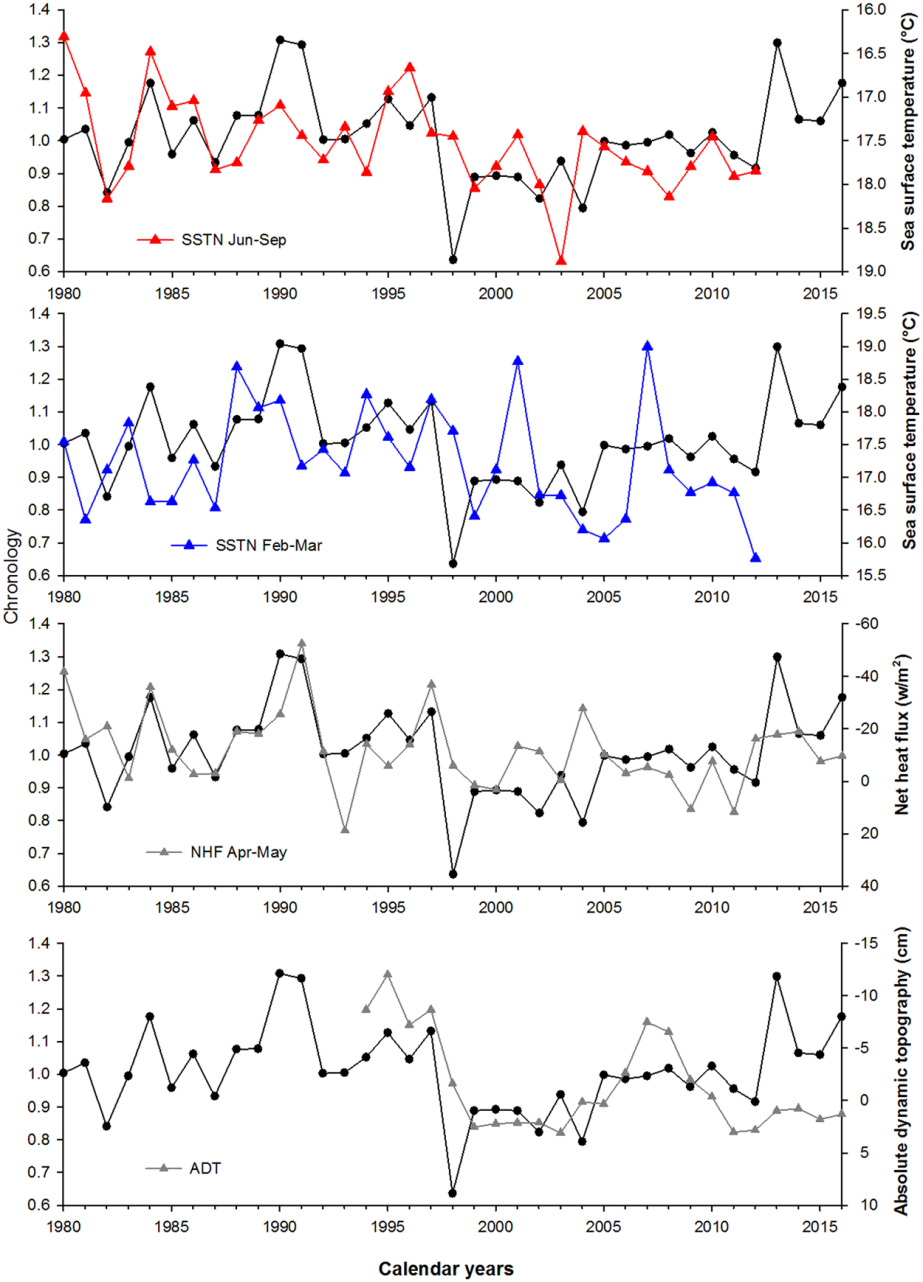
Time series data. Time series of environmental variables (coloured or grey) averaged over periods when Spearman correlation has been found significantly correlated with the Istria chronology (from top to bottom): sea surface temperature from NEMOMED12 (SSTN June-September), sea surface temperature from NEMOMED12 (SSTN February-March), downward net heat flux from ERA (NHF April-May), and absolute dynamic topography ADT October-September (yearly average). The chronology is displayed in black in all panels.

In addition to temperature, the chronology also relates to the Absolute Dynamic Topography (ADT) variable as an indicator of the Adriatic-Ionian Bimodal Oscillating System. Significant negative correlations (p < 0.05) were found between the chronology and ADT values for October, December, and February of the growth year (r values between −0.46 and −0.42, p values between 0.025 and 0.045, Fig. 3). Correlations are also significantly negative for the ADT annual averages (October-September, r = −0.43, p = 0.04). These negative correlations suggest that anticyclonic BiOS regimes are favourable for *G*. *pilosa* growth in the northern Adriatic Sea. Notably multi-month averages for other variables correlate relatively strongly with the bivalve chronology including June-August average sea surface temperature from NEMOMED12 (r = −0.537, p = 0.001), February-March average sea surface temperature from NEMOMED12 (r = 0.381, p = 0.029) and April-May average downward net heat flux from ERA (r = -0.393, p = 0.016) (Fig. 4). A multiple stepwise regression (p < 0.05 to enter), that includes June-August average sea surface temperature, February-March average sea surface temperature, April-May average downward net heat flux, and October-September ADT, finds that ADT (r = −0.62; p = 0.004) is the only significant predictor of chronology growth. A potential complication of this result is that both time series contain first-order autocorrelation, which could inflate the significance of the relationship. To address this issue, we removed autocorrelation from the chronology and ADT and repeated the regression, which remained significant (r = −0.47; p = 0.04). Thus, even with this “conservative” analysis in which important decadal-scale variability had been removed, the relationship between chronology and ADT was still robust.

## Discussion

### Chronology construction and potential for network development

Maximal longevity of *G*. *pilosa* individuals analysed in this study is somewhat longer than that noted for specimens from the Pašman Channel in the middle Adriatic Sea (72 vs. 69 years)^29^. At both sites, a SCUBA diver targeted larger sized shells, and the largest of these were analysed in this study with the goal of maximizing chronology length. These two populations are in close geographical proximity, and samples over this limited area are probably not fully representative of *G*. *pilosa* longevity. At both sites, however, the presence of older individuals (>60 year) suggests that longer chronologies are possible. Dead-collected individuals could theoretically be used to further extend chronologies back in time given the strength of the synchronous growth signals and possible overlap among samples by as much as forty or fifty years. Indeed, such an approach has been successfully applied in other bivalve species^8,30,31^ Given that this species inhabits other parts of the Mediterranean^32,33^, networks of chronologies may be possible throughout this semi-enclosed basin, which would serve to identify better regional-scale climate signals by reducing individual-site, local “noise”. For example, along the northeastern Pacific coast, the mean of three Pacific geoduck (*Panopea generosa*) chronologies separated by as much as 700 km strongly reflected regional and basin-wide water temperature signals. Indeed, the mean of these three chronologies explained almost 70% of the variance in British Columbia, Canada sea surface temperatures over a 60 yr interval^34^. In the North Sea, coherent signal has been found across a network of five *Arctica islandica* bivalve chronologies separated by up to 80 km^35^, underscoring the potential for capturing regional- to basin-wide signals. On even broader scales in the North Atlantic spanning >500 km, elements of coherence have been documented in the growth increment chronologies of *Glycymeris glycymeris* and *Arctica islandica*^36^. Thus, such an amplification of regional environmental signals may also be possible in the Mediterranean region.

### Correlations with environmental variables

Of the variables considered in this study, ADT was most strongly and consistently related to bivalve growth, underscoring the importance of BiOS regimes. Positive ADT values indicate a cyclonic BiOS regime in the northern Ionian Sea^18^ and the inflow of the ultraoligotrophic Eastern Mediterranean waters to the Adriatic, which are characterized by lower nutrient content as well as lower primary production and biological activity. These regime shifts occur on a decadal timescale and require up to a few years to make a full transition in the Adriatic^20,23,37^. Correlations with the bivalve chronology are negative through the year, but are strongest during late autumn and winter (October-February) when the inflow of saline waters to the Adriatic through the Otranto Strait is the largest^38^.

Yet, beyond the circulation patterns induced by BiOS regimes, sea water temperature may have at least some direct effect on growth. For example, February and March are generally characterized by minimum temperatures of 7–11 °C^39^, which are the lowest in the Mediterranean^27^. Positive correlations with winter sea water temperature suggest these lows may be limiting to *G*. *pilosa* growth such that favourable conditions are associated with milder winters. In contrast, the chronology is inversely related to summer sea temperature. Samples for this study were collected in shallow water (10 m) and are therefore strongly prone to the surface-driven heating. Ocean temperatures in the northern Adriatic may reach 30 °C in the summer months^39^, which could cause thermal stress and lower rates of growth. This is the first study to date to identify contrasting temperature responses across seasons for a bivalve chronology, underscoring the potentially complex climate-biology relationships in marine systems.

Although the chronology is related to indicators of ocean temperature and circulation, it does not correlate to indicators of freshwater inputs despite the shallow, coastal sampling location. These findings contrast with the classical model of the northern Adriatic in which biogeochemical processes can be strongly affected by the nutrient influx originating from rivers^26,40^ and local ocean dynamics^41^. Such signals might be weakened given that the northern Adriatic has experienced a decrease in anthropogenic forcing in the last two decades^42^, especially a decrease in nutrient loading from the rivers. Instead, our results indicate that more remote forcing by BiOS regimes is more important than these local, freshwater processes - at least in the eastern part of the northern Adriatic. This is consistent with previous findings for other open and coastal parts of the middle Adriatic^20,29^. Our findings are also supported by analysis of organic compounds in the northern Adriatic, where dissolved organic carbon concentration regimes have been found to qualitatively follow BiOS regimes in the last 25 years^28^.

### Reconstruction of environmental processes

Bivalve chronologies are proxies for the physical, biological, and chemical processes of marine and freshwater ecosystems^43^. Yet, most research to date has been directed toward long-living organisms at relatively high latitudes with an emphasis on hemispheric-scale processes such as the North Atlantic Oscillation^7,44^. Our results underscore the relevance of bivalve chronologies to local and regional scales, and also to temperate and semi-enclosed seas such as the Mediterranean. In this study, BiOS is a particularly strong driver, and one that overwhelms highly local influences of freshwater input. That is unexpected finding for the very shallow and closed areas such as the northern Adriatic, which are normally dependent on local heating/cooling or freshwater inputs^45–47^. Consequently, the Adriatic bivalve chronology may be used for reconstructing of BiOS-driven regimes in past climates in the Adriatic-Ionian region, especially if chronologies can be extended using dead-collected individuals^48^. These bivalves are likely to be the only means of developing exactly dated reconstructions of this relatively local yet highly biologically relevant climate process, underscoring the utility of the approach in quantifying fine-scale patterns of the climate system.

## Material and Methods

*Glycymeris pilosa* were live collected at 10–11 m depth by SCUBA from a coastal location in the North Adriatic (44°59′7.47″N, 13°44′19.22″E; Fig. 1). In order to obtain a sufficient number of individuals, sampling was conducted in May 2015, March 2016 and December 2016; immediately after collection bivalves were frozen. Divers targeted larger sized shells (>6 cm), from which the largest of these were chosen for this study. In the laboratory, shells were thawed and tissue was removed. *Glycymeris pilosa* has clearly visible annual growth increments in the hinge area^31^, which was cut from the rest of the shell and embedded in epoxy resin. Resin blocks containing the hinge were cut along axis of maximum growth, ground, and polished. Acetate peels were prepared after the polished surface had been etched in 0.1 M HCl for 2 minutes or 0.3 M HCl for 30 seconds, as has been previously applied to this species^29^. An Axio Lab A1 microscope equipped with Zeiss AxioCam ERc 5s camera was used to photograph acetate peels. Due to the size and magnification required, multiple photographs were taken of each sample and then stitched into a single panoramic using Image-Pro Premier software. A total of 66 shells was processed of which 30 were older than 35 years, had clearly delineated growth increments, and were used for chronology construction. The length of these shells ranged from 66.6 to 89.6 mm (80.52 ± 5.49 mm). According to oxygen isotope geochemistry^49^, *G*. *pilosa* in the North Adriatic forms an annual growth line in autumn. Therefore growth increment referred to as “2015” corresponds to shell growth from approximately October 2014 through September 2015.

The list-year method^50^ was used for visual crossdating, proceeding backward in time from the increment at the margin formed in the year of collection. Following visual crossdating, growth-increment widths were measured from the margin toward the center using Image-Pro Premier software. Program COFECHA^51,52^ was used for quantitative verification of crossdating. Each measurement time series was fitted with its own cubic smoothing spline of 15-year 50% frequency cutoff. Observed values were divided by those predicted to isolate high-frequency patterns of growth variability. Each detrended time series was then correlated with the mean of all others. Any unusually low correlation (p > 0.01) was visually re-inspected for possible dating errors. Series intercorrelation, the mean correlation between each individual time series and the average of all others, and mean sensitivity, an index of high frequency (year-to-year) variability between pairs of successive increments^53^, are reported.

The chronology was constructed in the software package ARSTAN^54^ using the original measurement time series. Each measurement time series was first power transformed^55^ and then detrended using a negative exponential function. Population-level signal strength in the chronology was assessed using the Expressed Population Signal (EPS). Although arbitrary, an EPS ~0.85 is considered the threshold at which the sample set adequately reflects the theoretical population from which it was drawn^56^. Only those portions of the chronology with at least 10 individuals were retained to ensure that this EPS threshold was exceeded.

Monthly series of sea surface temperature (SST), downward net heat flux (NHF), total precipitation (TP) and air temperature at 2 m (T2M) were extracted from gridded ERA-Interim reanalysis dataset^57^ between 1980 and 2016. Data were averaged across the study region, as delineated in red mesh polygon in Fig. 1. These data are downloadable at the ECMWF (European Centre for Middle-range Weather Forecast) website at http://www.ecmwf.int. Mean monthly discharge of the Po River (PO) were acquired from the Pontelagoscuro limnological station^25^ (Fig. 1).

Modelled monthly sea surface temperature (SSTN) and sea surface salinity (SSSN) at the uppermost model layer were averaged across the area (black mesh polygon in Fig. 1), as calculated by the NEMOMED12 ocean climate model and available at the Med-CORDEX webpage (https://www.medcordex.eu). Besides using ERA Interim products (here SST), we used SSTN from NEMOMED12 as the model provides the outputs at high resolution and also includes salinity (SSSN), which is not available by the ERA Interim. Yet, ERA Interim assimilates satellite products and therefore is the product of much higher reliability than the NEMOMED12 model variables. NEMOMED12 is an eddy-permitting oceanic regional model spanning the entire Mediterranean Sea and a buffer Atlantic zone off Gibraltar, with 50 unevenly spaced z levels in in the vertical dimension and a horizontal resolution of up to 8 km that is irregularly stretched to properly reproduce Gibraltar dynamics^58,59^. The model is run in hindcast mode and covers the ERA Interim period between 1980 and 2012. Annual and semi-annual cycles were removed from all environmental data sets.

Absolute dynamic topography multimission gridded data available at Copernicus – Marine Environment Monitoring Service were used for assessment of the Adriatic-Ionian Bimodal Oscillating System (BiOS) regimes. Annual and semi-annual cycles were removed from the data and then averaged for each month^20^. The difference between these values in the northern Ionian Sea and the central Ionian Sea (delineated here as the blue and red squares in Fig. 1, respectively) is defined as the absolute dynamic topography (ADT) variable. This difference has been used as an indicator of the BiOS^18,20,60^, or as an indicator of the cyclonic and anticyclonic circulation regime in the North Ionian.

Spearman’s rank correlation coefficients were used to relate chronologies with environmental data. Correlations were performed using monthly-averaged environmental variables given that climate conditions during specific months or seasons can strongly influence annual bivalve growth. Correlations were calculated between the chronology and gridded climate variables (T2M, SST, TP, ERA NHF, and NEMOMED12 values of SSTN and SSSN). Multiple stepwise linear regressions were also used to test whether the chronology was a function of a single environmental driver or a combination of drivers. Some variables contained autocorrelation, which can inflate levels of significance in correlation and regression analysis. To address this issue, all autocorrelation was removed from the chronology and environmental variables and the regression analysis was repeated. Although this may under-estimate the strength of the true relationship, it provides evidence that the relationship is robust.

## References

[1] Richardson AJ (2012). Climate change and marine life. Biology Lett..

[2] Marchitto TM, Jones GA, Goodfriend GA, Weidman CR (2000). Precise temporal correlation of Holocene mollusc shells using sclerochronology. Quaternary Res..

[3] Strom, A. Climate and fisheries in the Pacific Northwest: Historical perspectives from geoducks and early explorers. MSc thesis, University of Washington, Seattle (2003).

[4] Black BA (2016). The value of crossdating to retain high-frequency variability, climate signals, and extreme events in environmental proxies. Glob. Change Biol..

[5] Black BA (2014). Six centuries of variability and extremes in a coupled marine-terrestrial ecosystems. Science.

[6] Ambrose WG, Carroll ML, Greenacre M, Thorrold SR, McMahon KW (2006). Variations in *Serripes groenlandicus* (Bivalvia) growth in a Norwegian high-Arctic fjord: evidence for local- and large-scale climatic forcing. Glob. Change Biol..

[7] Butler PG, Wanamaker AD, Scourse JD, Richardson CA, Reynolds DJ (2013). Variability of marine climate on the North Icelandic Shelf in a 1357-year proxy archive based on growth increments in the bivalve *Arctica islandica*. Palaeogeogr. Palaeoclimatol. Palaeoecol..

[8] Wanamaker AD (2012). Surface changes in the North Atlantic meridional overturning circulation during the last millennium. Nat. Commun..

[9] Reynolds DJ (2016). Annually resolved North Atlantic marine climate over the last millennium. Nat. Commun..

[10] Schroeder K (2017). Rapid response to climate change in a marginal sea. Sci. Rep..

[11] Halpern BS (2015). Spatial and temporal changes in cumulative human impacts on the world’s ocean. Nat. Commun..

[12] Piroddi C (2017). Historical changes of the Mediterranean Sea ecosystem: modelling the role and impact of primary productivity and fisheries changes over time. Sci. Rep..

[13] Béthoux JP (1999). The Mediterranean Sea: a miniature ocean for climatic and environmental studies and a key for the climatic functioning of the North Atlantic. Prog. Oceanogr..

[14] Robinson, A. R., Leslie, W. G., Theocharis, A. & Lascaratos, A. Mediterranean Sea circulation, in Encyclopedia of Ocean Sciences, edited by J. H. Steele, S. A. Thorpe, and K. K. Turekian, pp. 1689–1705, Elsevier, Amsterdam (2003).

[15] Waldman R (2017). Modeling the intense 2012–2013 dense water formation event in the northwestern Mediterranean Sea: Evaluation with an ensemble simulation approach. J. Geophys. Res. Oceans.

[16] Pujo-Pay M (2011). Integrated survey of elemental stoichiometry (C, N, P) from the western to eastern Mediterranean Sea. Biogeosciences.

[17] Mihanović H (2013). Exceptional dense water formation on the Adriatic shelf in the winter of 2012. Ocean Sci..

[18] Gačić M, Eusebi Borzelli GL, Civitarese G, Cardin V, Yari S (2010). Can internal processes sustain reversals of the ocean upper circulation? The Ionian Sea example. Geophys. Res. Lett..

[19] Buljan M (1953). Fluctuation of salinity in the Adriatic. Izvještaj Republičke Ribarstveno-biološke ekspedicije “Hvar” 1948–1949. Acta Adriat..

[20] Mihanović H, Vilibić I, Dunić N, Šepić J (2015). Mapping of decadal middle Adriatic oceanographic variability and its relation to the BiOS regime. J. Geophys. Res..

[21] Gačić M (2011). On the relationship between the decadal oscillations of the northern Ionian Sea and the salinity distributions in the eastern Mediterranean. J. Geophys. Res..

[22] Civitarese G, Gačić M, Lipizer M, Eusebi Borzelli GL (2010). On the impact of the Bimodal Oscillating System (BiOS) on the biogeochemistry and biology of the Adriatic and Ionian Seas (Eastern Mediterranean). Biogeosciences.

[23] Batistić M, Garić R, Molinero JC (2014). Interannual variations in Adriatic Sea zoo- plankton mirror shifts in circulation regimes in the Ionian Sea. Climate Res..

[24] Vilibić I (2016). Hydrographic conditions driving sardine and anchovy populations in a land-locked sea. Mar. Med. Sci..

[25] Raicich F (1996). On the fresh water balance of the Adriatic Sea. J. Mar. Syst..

[26] Franco P, Michelato A (1992). Northern Adriatic Sea: oceanography of the basin proper and of the western coastal zone. Sci. Total Environ..

[27] Artegiani A (1997). The Adriatic Sea general circulation. Part I. Air-sea interaction and water mass structure. J. Phys. Oceanogr..

[28] Dautović J, Vojvodić V, Tepić N, Ćosović B, Ciglenečki I (2017). Dissolved organic carbon as potential indicator of global change: A long-term investigation in the northern Adriatic. Sci. Total Environ..

[29] Peharda M, Black BA, Purroy A, Mihanović H (2016). The bivalve *Glycymeris pilosa* as a multidecadal environmental archive for the Adriatic and Mediterranean Seas. Mar. Environ. Res..

[30] Featherstone AM, Butler PG, Peharda M, Chauvaud L, Thébault JZ (2017). Influence of riverine input on the growth of *Glycymeris glycymeris* in the Bay of Brest, North-West France. PLoS ONE.

[31] Reynolds DJ (2017). Reconstructing past seasonal to multicentannial-scale variability in the NE Atlantic ocean using the long-lived marine bivalve mollusc *Glycymeris glycymeris*. Paleoceanography.

[32] Poppe, T., Goto, Y. European seashells Vol. II. (Scaphopoda, Bivalvia, Cephalopoda). Wiesbaden: Verlag Christa Hemmen (1993).

[33] Purroy A (2016). Combined use of morphological and molecular tools to resolve species mis-identification in the Bivalvia – the case of *Glycymeris glycymeris* and *G*. *pilosa*. PLoS ONE.

[34] Black BA, Copenheaver CA, Frank DC, Stuckey MJ, Kormanyos RE (2009). Multi-proxy reconstructions of northeastern Pacific sea surface temperature data from trees and Pacific geoduck. Palaeogeogr. Palaeoclimatol. Palaeoecol..

[35] Butler PG (2009). Accurate increment identification and the spatial extent of the common signal in five *Arctica islandica* chronologies from the Fladen Ground, northern North Sea. Paleoceanography.

[36] Reynolds DJ (2017). Reconstructing North Atlantic marine climate variability using an absolutely-dated sclerochronological network. Palaeogeogr. Palaeoclimatol. Palaeoecol..

[37] Matić F (2017). Oscillating Adriatic temperature and salinity regimes mapped using the Self-Organizing Maps method. Cont. Shelf Res..

[38] Gačić (1996). Thermohaline properties and circulation in the Otranto Strait. Bulletin de l’Institut océaographique, Monaco, n° special.

[39] Supić N, Grbec B, Vilibić I, Ivančić I (2004). Long-term changes in hydrographic conditions in northern Adriatic and its relationship to hydrological and atmospheric processes. Ann. Geophys..

[40] Degobbis D (2000). Long-term changes in the northern Adriatic ecosystem related to anthropogenic eutrophication. Int. J. Environ. Pollut..

[41] Kraus R, Supić N, Precali R (2016). Factors favouring phytoplankton blooms in the northern Adriatic: towards the northern Adriatic empirical ecological model. Ocean Sci..

[42] Djakovac T, Degobbis D, Supić N, Precali R (2012). Marked reduction of eutrophication pressure in the northeastern Adriatic in the period 2000-2009. Estuar. Coast. Shelf Sci..

[43] Schöne BR (2003). North Atlantic Oscillation dynamics recorded in shells of a long-lived bivalve mollusk. Geology.

[44] Lohmann G, Schöne BR (2013). Climate signatures on decadal to interdecadal time scales as obtained from mollusk shells (*Arctica islandica*) from Iceland. Palaeogeogr. Palaeoclimatol. Palaeoecol..

[45] Dekker R, Beukema JJ (1999). Relations of summer and winter temperatures with dynamics and growth of two bivalves, *Tellina tenuis* and *Abra tenuis*, on the northern edge of their intertidal distribution. J. Sea Res..

[46] Schöne BR, Lega J, Flessa KW, Goodwin DH, Dettman DL (2002). Reconstructing daily temperatures from growth rates of the intertidal bivalve mollusk *Chione cortezi* (northern Gulf of California, Mexico). Palaeogeogr. Palaeoclimatol. Palaeoecol..

[47] Hallmann N (2011). An improved understanding of the Alaska Coastal Current: The application of a bivalve growth-temperature model to reconstruct freshwater-influenced paleoenvironments. Palaios.

[48] Scourse J (2006). First cross-matched floating chronology from the marine fossil record: data from growth lines of the long-lived bivalve mollusc *Arctica islandica*. Holocene.

[49] Peharda, M. *et al*. Contrasting shell growth strategies in two Mediterranean bivalves revealed by oxygen isotope geochemistry: the case of *Pecten jacobaeus* and *Glycymeris pilosa*. *Chem*. *Geol*. 10.1016/j.chemgeo.2017.09.029 (2018).

[50] Yamaguchi DK (1991). A simple method for cross-dating increment cores from living trees. Can. J. Forest Res..

[51] Holmes RL (1983). Computer-assisted quality control in tree-ring dating and measurements. Tree-Ring Bull..

[52] Grissino-Mayer HD (2001). Evaluating crossdating accuracy: a manual and tutorial for the computer program COFECHA. Tree Ring Res..

[53] Fritts, H.C. *Tree Rings and Climate*. Academic Press, New York, 584 (1976).

[54] Cook, E. R. & Holmes, R. L. Guide for computer program ARSTAN. *In The International Tree-Ring Data Bank Program Library Version 2*.*0 User’s Manu**al* (eds Grissino Mayer, H. D., Holmes, R. L. & Fritts, H. C.). University of Arizona, Tucson, Ariz. (1996).

[55] Cook ER, Peters K (1997). Calculating unbiased tree-ring indices for the study of climatic and environmental change. Holocene.

[56] Wigley TML, Briffa KR, Jones PD (1984). On the average value of correlated time-series, with applications in dendroclimatology and hydrometeorology. J. Clim. Appl. Meteorol..

[57] Berrisford, P. *et al*. The ERA-Interim archive Version 2.0. ERA Report Series 1, ECMWF, Reading, available at https://www.ecmwf.int/sites/default/files/elibrary/2011/8174-era-interim-archive-version-20.pdf (2011).

[58] Lebeaupin Brossier C, Béranger K, Deltel C, Drobinski P (2011). The Mediterranean response to different space–time resolution atmosphericforcings using perpetual mode sensitivity simulations. Ocean Model..

[59] Lebeaupin Brossier C, Béranger K, Drobinski P (2012). Sensitivity of the northwestern Mediterranean Sea coastal and thermohaline circulations simulated by the 1/12°-resolution ocean model NEMO-MED12 to the spatial and temporal resolution of atmospheric forcing. Ocean Model..

[60] Gačić M (2014). Extreme winter 2012 in the Adriatic: an example of climatic effect on the BiOS rhythm. Ocean Sci..

